# The Role of Auxin-Cytokinin Antagonism in Plant-Pathogen Interactions

**DOI:** 10.1371/journal.ppat.1003026

**Published:** 2012-11-29

**Authors:** Muhammad Naseem, Thomas Dandekar

**Affiliations:** Department of Bioinformatics, University of Würzburg, Würzburg, Germany; The University of North Carolina at Chapel Hill, United States of America

## Auxin-Cytokinin Antagonism Is Important Both for Development and Immunity

It has been several decades since Skoog and Miller described the contrasting behavior of auxin and cytokinin in promoting the growth of root and shoot, respectively [1]. In recent years, a lot of progress has been made in understanding the regulation of stem cell niche and cell fate in both shoot and root apical meristems. Developmental processes such as the maintenance of root meristems [2], lateral root formation [3], leaf position determination [4], and *de novo* auxin-induced organogenesis [5] are fine-tuned by the mutual interactions between auxin and cytokinin. Auxin exerts its inhibition on cytokinin on several levels; mechanisms range from its biosynthesis to the suppression of its signaling [6]. Reciprocally, cytokinin antagonistically impacts the flux, distribution, and signaling of auxin [7]. Antagonism between auxin and cytokinin is not the only type of interaction that governs developmental outputs in plants. Rather, synergistic interaction between auxin and cytokinins also exists in processes such as nodule organogenesis [8], light-mediated leaf initiation, and organ positioning [9]. With the majority of previous studies focusing on these hormones in development, auxin-cytokinin interplay has not been extensively analyzed in the context of plant immunity. Mutual interactions between stress-specific hormones such as salicylic acid and jasmonic acid/ethylene (SA-JA/ET) are regarded as the central backbone of the immunity [10]. However, growth-promoting hormones (auxin, cytokinins, gibberellic acid, and abscisic acid) either inhibit or potentiate this balance in mediating the protection or susceptibility of the plant against the invading pathogen [10], [11]. For a comprehensive understanding of hormonal crosstalk in disease, a systems-biological perspective is critical, as plant hormones act in concert [11]. We focus on recent progress regarding the individual effects of auxin and cytokinins and their combined effect on immune dynamics in plant-pathogen systems.

## Auxin Promotes Susceptibility of *Arabidopsis* against Infection with *Pseudomonas syringae* pv. *tomato* DC3000

**Figure 1:**
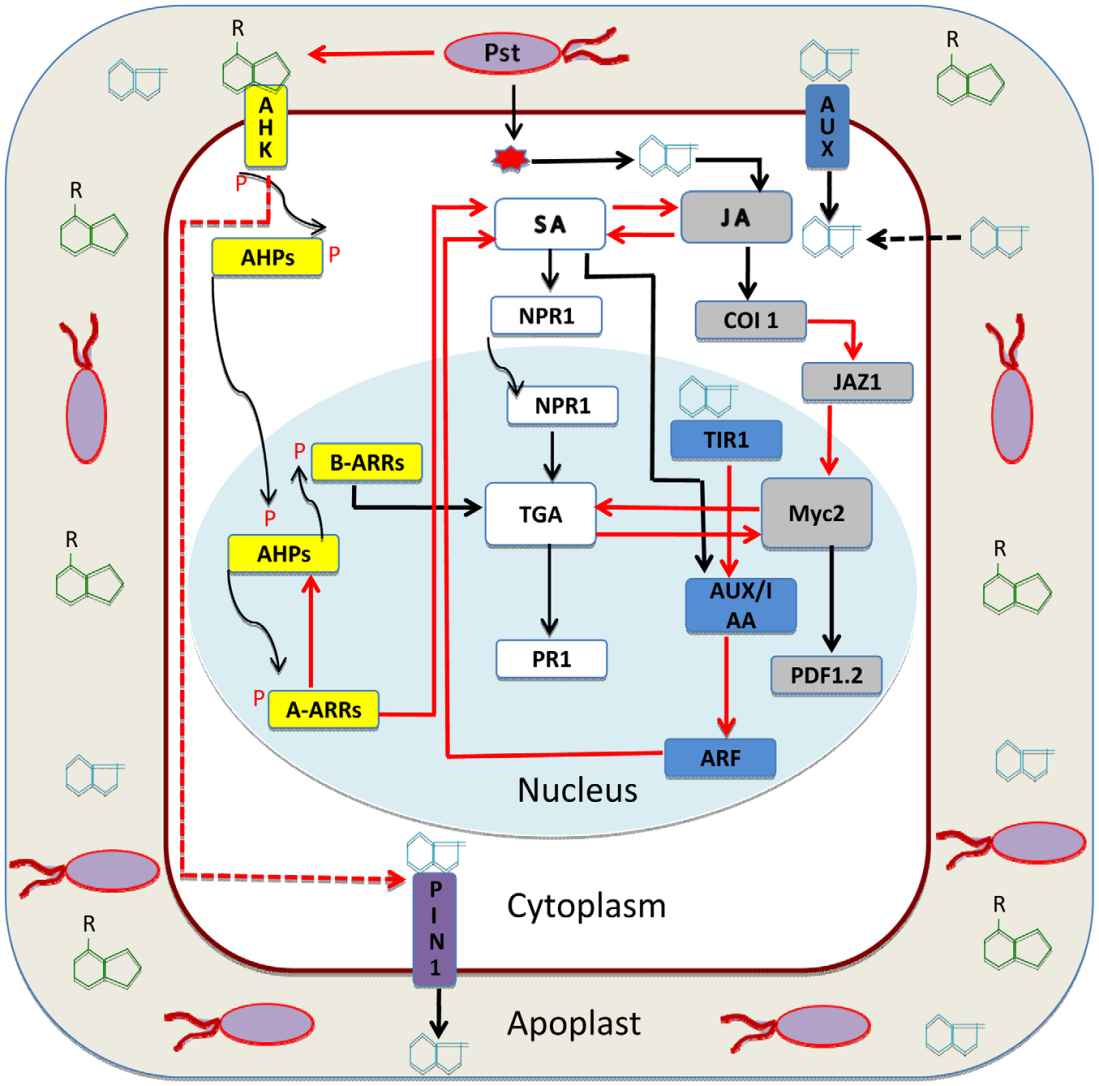
Auxin-cytokinin interaction in plant immunity. *Pseudomonas syringae* pv. *tomato* DC3000 (*Pto*) resides in the apoplast of adjacent *Arabidopsis* cells [30]. Besides other substances, the apoplast also contains various sugars, ions, and free hormones cytokinin and auxin (green and light blue chemical structures). *Pto* injects effectors via TTSS (Type III secretion system) into the plant cell [11]. This causes higher auxin accumulation in the cytoplasm. Auxin also diffuses directly from the apoplast or is taken into the cytoplasm by AUX1 (auxin influx transporter) [13]. Excess of auxin releases ARF (auxin response factor) from AUX/IAA (auxin repressor) and keeps auxin responses derepressed [16]. Auxin crosstalk enhances synergistically the JA pathway (gray boxes) at the level of JA biosynthesis and downstream signaling to enhance PDF1.2 (marker for JA/ET pathway [13]). Auxin represses the SA pathway (white boxes) to cause susceptibility of *Arabidopsis* against the infection of *Pto* [18]. During the course of infection, *Pto* decreases the level of cytokinin (green structures in apoplast) [11]. Cytokinins are perceived by AHKs (*Arabidopsis* histidine kinase) and transduced by a two-component phosphorelay system (yellow boxes); the signal of phosphorylation is transduced from receptor to AHPs (*Arabidopsis* histidine phosphotransfer protein) in the cytoplasm [6]. Subsequently, type-B ARRs (positive regulator of cytokinin response) are phosphorylated, and they in turn activate the expression of type-A ARRs (repressor of cytokinin signaling) [28]. Cytokinin activates the SA pathway (white boxes) at the level of TGA3 via type-B ARR2 in an NPR1-dependent manner [21]. In a negative feedback loop, type-A ARRs also inhibit cytokinin responses [28]. Cytokinin also inhibits auxin transport by keeping PIN1 auxin efflux transporters arrested and thus causes imbalance in auxin flux and distribution inside the tissue [7]. Auxin and cytokinin, besides mutual inhibition, contribute differentially to the SA-JA/ET backbone of plant defense against pathogens [11].

The importance of the SA-JA/ET core defense signaling in plant-pathogen interactions has long been established [12]. However, our understanding of the influence growth-promoting hormones exert on modulating the central defense pathways is still lagging behind. A focus on this aspect of plant immunity will disclose important biological inferences concerning the trade-off between defense and development in plants. The impact of auxin has already been analyzed with respect to immune dynamics of plant-pathogen interactions [13]. During the course of infection, *Pseudomonas syringae* pv. *tomato* DC3000 (*Pto*) causes a *de novo* increase of auxin biosynthesis in *Arabidopsis* [14] and therefore ensures optimal bacterial multiplication. Depriving bacteria from effector-secretion capability (Type III secretion system, TTSS mutants) reduces their *in planta* bacterial growth. However, the transgenic expression of the *AvrRpt2* (bacterial effector protein) gene in *Arabidopsis* restores optimal growth of the *Pto* strain defective in TTSS, thereby linking the effector and auxin (Figure 1) in mediating susceptibility of the host [15]. Likewise, the inhibition of auxin receptor TIR1 (Figure 1) by overexpression of miR393 (suppressor of auxin signaling) results in the protection of *Arabidopsis* against infection by *Pto* [16]. SA-mediated stabilization of the auxin signaling repressor AUX/IAA (Figure 1) renders the plant host resistant to biotrophic infections (pathogens that feed only on live host tissue [17]). Reciprocally, auxin inhibits the SA responses [18], which in turn partly strengthens the role of the JA pathway in immunity and thus keeps the host susceptible to infection by *Pto*. The underlying mechanism by which auxin promotes the susceptibility of the host is multilayered. On the one hand, *Pto* counter-regulates SA responses by producing higher *in planta* auxin levels (Figure 1). Moreover, increased auxin levels not only increase the concentration of JA but also enhance JA signaling [13], which in turn weakens the plant defense against biotrophic pathogens. On the other hand, the conjugated forms of auxin also promote bacterial growth in the infected *Arabidopsis* plants by directly regulating the transcription of the virulence genes in *Pto* [19]. Thus, auxin plays a pivotal role in shaping the hormonal dynamics of *Pto-Arabidopsis* interactions in favor of bacterial growth as well as efficient disease development.

## Cytokinin Confers Protection to *Arabidopsis* against Infection with *Pto*

In contrast to auxin, the role of cytokinin has not been systematically analyzed. Previously, cytokinins were believed to be instrumental in mediating host susceptibility against fungal biotrophs by generating a green island around the infection zones [20]. Moreover, the concerted action of cytokinins together with auxin in *Agrobacterium tumefaciens–*driven crown gall (or leafy gall: pathogen-induced plant tumors) formation has been reported [21], [22]. However until recently, their impact on the model interaction between *Pto* and *Arabidopsis* has not been emphasized. High levels of cytokinins were found to increase the resistance of plants to some viral pathogens and herbivores [23]. Similarly, the expression of the *PR1* gene was shown as upregulated in the presence of enhanced levels of plant cytokinin, while overexpression of cytokinin oxidase caused its repression in *Arabidopsis* [24]. Recently, transgenic overexpression of isopentenyltransferase enzyme genes (*AtIPT1*, *2*, *5*, and *7*) increased *Arabidopsis* resistance to *Pto* [25]. At the same time, overexpression of genes encoding cytokinin oxidase *(AtCKX4)* enhanced susceptibility of *Arabidopsis* to infection by pathogens [21], [25]. The research group attributed this cytokinin-mediated resistance to SA-dependent mechanisms. They described that ARR2 (type-B ARR, a positive regulator of cytokinin signaling) interacts with TGA3 (a transcription factor involved in inducing SA-responsive genes) in the regulation of disease marker gene *PR1* against biotrophic infections in plants (Figure 1). *PR1* is furthermore controlled by the SA transcriptional activator/receptor [26] called NPR1 (nonexpressor of pathogenesis-related gene 1). However, Grosskinsky et al. [27] described an enhanced SA-independent but phytoalexin-dependent (capsidiol and scopoletin) mechanism for the *Pseudomonas syringae* pv. tabaci and tobacco interaction. In addition to the enhanced resistance against *Pto*, cytokinin has also been shown to protect *Arabidopsis* against the necrotrophic pathogen (a pathogen that feeds on dead host tissue) *Alternaria brassicicola* (see Figure S1 in [25]). Similarly, cytokinin-producing and non-cytokinin-producing pathogens have their own peculiar modes of interaction with their corresponding hosts [25]. Argueso et al. [28] demonstrated a dose-dependent response to cytokinin, such that higher levels of the hormone decreased *Arabidopsis* susceptibility to infection by *Hyaloperonospora arabidopsidis* (*Hpa*) Noco2. They also implicated the negative regulators (type-A ARRs) of cytokinin signaling as acting in a feedback loop (Figure 1). Very recently, Naseem et al. [11] further explored the interaction between cytokinin and SA signaling. In SA-biosynthesis mutants (*sid2* mutants), the exogenous application of cytokinin partially inhibited the growth of the pathogen. However in the wild type *Arabidopsis* plants, the enhanced levels of cytokinin gave rise to more protection than that observed in the *sid2* mutants. This strongly suggests that cytokinin signaling enhances the contribution of SA-mediated immunity in hormone disease networks. The SA-dependent response to cytokinin application is not limited to a few marker genes, but seems to be a genome-wide transcriptional reprogramming [11]. Moreover, the increased phytoalexin accumulation observed during higher levels of cytokinin in the plant has additional implications in promoting resistance against infection with *Pto.*

## Interplay between Auxin and Cytokinin Modulates the *Pto*-*Arabidopsis* Interaction

The individual roles of auxin and cytokinin vary among plant-pathogen systems [16], [17], [21], [25]. To assess the impact of the auxin-cytokinin antagonism on plant immune defense, we have focused on a single model plant-pathogen system: *Arabidopsis* infected by *Pto* (Figure 1). Auxin enhances susceptibility, and there is repression of *PR1* during elevated plant auxin levels [13]. Elevated cytokinin levels mediate resistance and induction of *PR1* [11], [21], [25]. This suggests a plausible auxin-cytokinin antagonism during infection with *Pto* in *Arabidopsis*. Recent studies regarding hormonal implications in plant immunity have highlighted that the roles of auxin and cytokinin are independent [12]. However to analyze auxin-cytokinin antagonism, the impact of both hormones in plant immunity should be investigated in concert. A recent report by Robert-Salinaintz et al. [18] has demonstrated that during the course of infection, *Pto* enhances *de novo* auxin accumulation, whereas the level of cytokinin decreases as compared to the basal levels. This increased *in planta* auxin concentration in *Pto*-infected leaves is believed to be due to the presence of effector protein(s), since a *Pto* TTSS mutant strain failed to induce this response during infection [14], [15]. However, no increase in cytokinin concentration post–pathogen infection was observed under the same conditions [11], [18]. In order to keep the host susceptible, pathogen infection appears to cause a decrease in *t*-zeatin levels during *Pto-Arabidopsis* interaction [11]. This is crucial for the pathogen, as cytokinin catabolism (overexpression of *CKX4* gene [25]) favors fast bacterial growth. Genome-wide transcriptional studies also support the upregulation of auxin biosynthesis genes during infection with *Pto* in *Arabidopsis*. The genes encoding enzymes of cytokinin biosynthesis were found to be downregulated, while those of cytokinin catabolism were induced [29]. These and similar data [11] support the opposing roles of auxin and cytokinin in shaping immune dynamics during the interaction between *Pto* and *Arabidopsis.*

We propose a working model for the auxin-cytokinin antagonism (Figure 1): To cause susceptibility, *Pto* enhances the *in planta* level of auxin, which attenuates the SA responses and hence decreases resistance against infection by a pathogen. Additionally, auxin downsizes the pool of cytokinin, which could also lead to increased susceptibility. Cytokinin, when exogenously applied or transgenically enhanced, expedites SA responses in the host [25]. It thus diminishes the anticipated auxin-based susceptibility. Cytokinin is known to be instrumental in inhibiting auxin transport [6], [7], and auxin inhibition has been linked to increased resistance to infection by many pathogens, including *Pto* [13]. The exact molecular mechanism whereby auxin-cytokinin antagonism governs the outcome of *Pto* and *Arabidopsis* interaction is still not well understood. One plausible explanation (Figure 1) for the reduced auxin signaling could be similar to the one demonstrated for apical root meristem. There cytokinin invokes type-B ARRs [21] to stabilize the auxin repressor AUX/IAA [2], thus suppressing auxin responses [18], while auxin may also cause repression of cytokinin responses by activating negative regulators (type-A ARRs [6]) of cytokinin signaling [28]. These and similar hypotheses need to be addressed with detailed experiments in the context of hormone disease networks.

## Concluding Remarks

Here we highlight the auxin-cytokinin antagonism that occurs as part of a complex hormonal interplay and exerts a critical influence on the core SA-JA/ET plant immunity pathways [12]. Auxin inhibits SA responses and thus indirectly promotes the contribution of JA signaling in immunity. In contrast, cytokinin reinforces SA responses during plant defense against *Pto*. To address this more closely, we advocate a systems-biology approach to analyze the impact of interactions between auxin and cytokinin in plant immunity [11]. Both modeling and experimental data analysis are currently converging to describe plant immunity as a system output governed by a finely tuned balance between auxin and cytokinin. It is the interaction of different hormonal networks that modulates plant immunity, rather than just the contribution of auxin and cytokinin. Auxin-cytokinin interaction is crucial in defining the dynamics of growth and development. However, recent work shows that this interaction should be equally important in plant immunity networks.

## References

[1] Skoog F, Miller CO (1957) Chemical regulation of growth and organ formation in plant tissues cultured in vitro. Symp Soc Exp Biol 54: 118–130. 13486467

[2] Dello Ioio R, Nakamura K, Moubayidin L, Perilli S, Taniguchi M, et al (2008) A genetic framework for the control of cell division and differentiation in the root meristem. Science 322: 1380–1384. 19039136 10.1126/science.1164147

[3] Laplaze L, Benkova E, Casimiro I, Maes L, Vanneste S, et al (2007) Cytokinins act directly on lateral root founder cells to inhibit root initiation. Plant Cell 12: 3889–3900. 10.1105/tpc.107.055863 PMC2217640 18065686

[4] Shimizu-Sato S, Tanaka M, Mori H (2009) Auxin-cytokinin interactions in the control of shoot branching. Plant Mol Biol 69: 429–435. 18974937 10.1007/s11103-008-9416-3

[5] Pernisová M, Klíma P, Horák J, Válková M, Malbeck J, et al (2009) Cytokinins modulate auxin-induced organogenesis in plants via regulation of the auxin efflux. Proc Natl Acad Sci U S A 106: 3609–3614. 19211794 10.1073/pnas.0811539106 PMC2640219

[6] Moubayidin L, Di Mambro R, Sabatini S (2009) Cytokinin-auxin crosstalk. Trends Plant Sci 14: 557–562. 19734082 10.1016/j.tplants.2009.06.010

[7] Stepanova AN, Alonso JM (2011) Bypassing transcription: a shortcut in cytokinin-auxin interactions. Dev Cell 4: 608–610. 10.1016/j.devcel.2011.09.016 22014520

[8] Hwang I, Sheen J, Muller B (2012) Cytokinin signaling networks. Annu Rev Plant Biol 63: 353–380. 22554243 10.1146/annurev-arplant-042811-105503

[9] Yoshida S, Mandel T, Kuhlemeier C (2011) Stem cell activation by light guides plant organogenesis. Genes Dev 13: 1439–1450. 10.1101/gad.631211 PMC3134086 21724835

[10] Pieterse CM, van der Does D, Zamioudis C, Leon-Reyes A, van Wees SC (2012) Hormonal modulation of plant immunity. Annu Rev Cell Dev Biol [Epub ahead of print]. 10.1146/annurev-cellbio-092910-154055 22559264

[11] Naseem M, Philippi N, Hussain A, Wangorsch G, Ahmed N, et al (2012) Integrated systems view on networking by hormones in Arabidopsis immunity reveals multiple crosstalk for cytokinin. Plant Cell 24: 1793–1814. 22643121 10.1105/tpc.112.098335 PMC3442570

[12] Robert-Seilaniantz A, Grant M, Jones JDG (2011) Hormone crosstalk in plant disease and defense: more than just jasmonate-salicylate antagonism. Annu Rev Phytopathol 49: 317–343. 21663438 10.1146/annurev-phyto-073009-114447

[13] Kazan K, Manners JM (2009) Linking development to defense: auxin in plant-pathogen interactions. Trends Plant Sci 7: 373–382. 10.1016/j.tplants.2009.04.005 19559643

[14] Chen Z, Agnew JL, Cohen JD, He P, Shan L, et al (2007) Pseudomonas syringae type III effector AvrRpt2 alters Arabidopsis thaliana auxin physiology. Proc Natl Acad Sci U S A 104: 20131–20136. 18056646 10.1073/pnas.0704901104 PMC2148434

[15] Chen Z, Kloek AP, Cuzick A, Moeder W, Tang D, et al (2004) The Pseudomonas syringae type III effector AvrRpt2 functions downstream or independently of SA to promote virulence on Arabidopsis thaliana. Plant J 37: 494–504. 14756766 10.1111/j.1365-313x.2003.01984.x

[16] Navarro L, Dunoyer P, Jay F, Arnold B, Dharmasiri N, et al (2006) A plant miRNA contributes to antibacterial resistance by repressing auxin signaling. Science 312: 436–439. 16627744 10.1126/science.1126088

[17] Wang D, Mukhtar KP, Culler AH, Dong X (2007) Salicylic acid inhibits pathogen growth in plants through repression of the auxin signaling pathway. Curr Biol 17: 1784–1790. 17919906 10.1016/j.cub.2007.09.025

[18] Robert-Seilaniantz A, MacLean D, Jikumaru Y, Hill L, Yamaguchi S, et al (2011) The microRNA miR393 re-directs secondary metabolite biosynthesis: away from camalexin and towards glucosinolates. Plant J 67: 218–231. 21457368 10.1111/j.1365-313X.2011.04591.x

[19] Gonzalez-Lamothe R, Oirdi ME, Brission N, Bauarab K (2012) The conjugated auxin indole-3-acetic acid-aspartic acid promotes plant disease development. Plant Cell 24: 762–777. 22374398 10.1105/tpc.111.095190 PMC3315245

[20] Walters DR, McRoberts N (2006) Plants and biotrophs: a pivotal role for cytokinins? Trends Plant Sci 11: 581–586. 17092762 10.1016/j.tplants.2006.10.003

[21] Choi J, Choi D, Lee S, Ryu CM, Hwang I (2011) Cytokinins and plant immunity: old foes or new friends? Trends Plant Sci 7: 388–394. 10.1016/j.tplants.2011.03.003 21470894

[22] Pertry I, Václavíková K, Depuydt S, Galuszka P, Spíchal L, et al (2009) Identification of *Rhodococcus fascians* cytokinins and their modus operandi to reshape the plant. Proc Natl Acad Sci U S A 106: 929–934. 19129491 10.1073/pnas.0811683106 PMC2630087

[23] Ballaré CL (2011) Jasmonate-induced defenses: a tale of intelligence, collaborators and rascals. Trends Plant Sci 5: 249–257. 10.1016/j.tplants.2010.12.001 21216178

[24] Uchida N, Tasaka M (2010) Interactions between immune responses and morphological regulation in plant. J Exp Bot 61: 2539–2547. 20457577 10.1093/jxb/erq126

[25] Choi J, Huh SU, Kojima M, Sakakibara H, Paek KH, et al (2010) The cytokinin-activated transcription factor ARR2 promotes plant immunity via TGA3/NPR-1-dependent salicylic acid signalling in Arabidopsis. Dev Cell 19: 284–295. 20708590 10.1016/j.devcel.2010.07.011

[26] Wu Y, Zhang D, Chu JY, Boyle P, Wang Y, et al (2012) The Arabidopsis NPR1 protein is a receptor for the plant defense hormone salicylic acid. Cell Rep [Epub ahead of print]. 10.1016/j.celrep.2012.05.008 22813739

[27] Grosskinsky DK, Naseem M, Abdelmohsen UR, Plickert N, Engelkeet T, et al (2011) Cytokinins mediate resistance against Pseudomonas syringae in tobacco through increased antimicrobial phytoalexin synthesis independent of salicylic acid signalling. Plant Physiol 157: 815–830. 21813654 10.1104/pp.111.182931 PMC3192561

[28] Argueso CT, Ferreira FJ, Epple P, To JPC, Hutchison CE, et al (2012) Two-component elements mediate interactions between cytokinin and salicylic acid in plant immunity. PLoS Genet 8: e1002448 doi:10.1371/journal.pgen.1002448. 22291601 10.1371/journal.pgen.1002448 PMC3266875

[29] Thilmony R, Underwood W, He SY (2006) Genome-wide transcriptional analysis of the Arabidopsis thaliana interaction with the plant pathogen *Pseudomonas syringae* pv. tomato DC3000 and the human pathogen *Escherichia coli* O157:H7. Plant J 46: 34–53. 16553894 10.1111/j.1365-313X.2006.02725.x

[30] Rico A, McCraw SL, Preston GM (2011) The metabolic interface between *Pseudomonas syringae* and plant cells. Curr Opin Microbiol 14: 31–38. 21236723 10.1016/j.mib.2010.12.008

